# Androgen Flare after LHRH Initiation Is the Side Effect That Makes Most of the Beneficial Effect When It Coincides with Radiation Therapy for Prostate Cancer

**DOI:** 10.3390/cancers14081959

**Published:** 2022-04-13

**Authors:** Nicola J. Nasser

**Affiliations:** 1 Department of Radiation Oncology, Albany Medical College, Albany, NY 12208, USA; nassern@amc.edu or nicola.nasser@gmail.com; 2 The Umbilicus Inc., Nonprofit Organization for Preserving Sexual Function of Individuals with Cancer Below the Umbilicus, New York, NY 10032, USA

**Keywords:** prostate cancer, hormonal therapy, radiation therapy, synthetic lethality, testosterone flare, mitotic catastrophe

## Abstract

**Simple Summary:**

Prostate cancer tumor growth is stimulated by androgens. Surgical castration or medical castration using long-acting luteinizing hormone-releasing hormone (LHRH) agonists or antagonists is the backbone of the treatments of metastatic disease. Treatment of locally advanced prostate cancer was accomplished with radiation therapy alone until multiple studies showed that combining radiation therapy with LHRH agonists results in significant survival benefit. While the goal of the use of LHRH agonists was to suppress testosterone levels during radiation, we show, through review of previous studies, that survival benefit was achieved only when LHRH was initiated during the course of radiation, and thus androgen flare during the first 1–3 weeks after the initiation of LHRH is most likely the reason for higher survival. Androgens drive tumor cells into mitosis, and mitotic death is the dominant mechanism of tumor cell kill by radiation.

**Abstract:**

Treatment of metastatic prostate cancer was historically performed via bilateral orchiectomy to achieve castration. An alternative to surgical castration is the administration of subcutaneous recombinant luteinizing hormone-releasing hormone (LHRH). LHRH causes the pituitary gland to produce luteinizing hormone (LH), which results in synthesis and secretion of testosterone from the testicles. When LHRH levels are continuously high, the pituitary gland stops producing LH, which results in reduced testosterone production by the testicles. Long-acting formulations of LHRH were developed, and its use replaced surgical orchiectomy in the vast majority of patients. Combining LHRH and radiation therapy was shown to increase survival of prostate cancer patients with locally advanced disease. Here, we present a hypothesis, and preliminary evidence based on previous randomized controlled trials, that androgen surge during radiation, rather than its suppression, could be responsible for the enhanced prostate cancer cell kill during radiation. Starting LHRH agonist on the first day of radiation therapy, as in the EORTC 22863 study, should be the standard of care when treating locally advanced prostate cancer. We are developing formulations of short-acting LHRH agonists that induce androgen flare, without subsequent androgen deprivation, which could open the door for an era in which locally advanced prostate cancer could be cured while patients maintain potency.

## 1. Introduction

In 1941, Charles Huggins and Clarence V. Hodges published a report on 8 patients with carcinoma of the prostate metastatic to bone who underwent bilateral orchiectomy [1]. Since then, castration became the main modality of treatment of metastatic prostate cancer. The first evidence for the existence of hypothalamic substances that controls the secretion of hormones from the anterior pituitary gland was reported by Saffran and Schally in 1955 [2], when they identified the corticotropin-releasing factor (CRF). The purification and identification of the LHRH sequence of 10 amino acids was reported by the group of Schally in 1971 [3,4]. The understanding of the mechanism of action of LHRH, and the development of long-acting agonists and antagonists to LHRH, resulted in a shift from surgical castration [5] to the use of long-acting LHRH agonists [6,7,8] and antagonists [9,10,11,12,13] to induce castrate androgen levels. LHRH causes the pituitary gland to produce luteinizing hormone (LH), which results in synthesis and secretion of testosterone from the testicles. When LHRH levels are continuously high, the pituitary gland stops producing LH, which results in reduced testosterone production by the testicles.

The seminal study by Bolla et al. [7] that compared external irradiation with external irradiation plus goserelin, an agonist analogue of gonadotropin-releasing hormone, showed survival benefit for adding hormonal therapy to radiation compared to radiation alone. Warde et al. randomized patients with locally advanced prostate cancer to ADT, versus radiation therapy and ADT (LHRH agonist used in 92% and orchiectomy in 8% of patients), and showed that the combined therapy resulted in better survival [14]. Thus, combining LHRH agonist with radiation for locally advanced prostate cancer results in a survival benefit, compared to either of the treatments alone. This means that there is more tumor cell kill when LHRH agonist is combined with radiation.

The current report aims to show that the beneficial part of combining LHRH and radiation is androgen flare and not androgen deprivation, and that androgen suppression could result in resistance to radiation and the need for dose escalation.

## 2. LHRH Agonists Result in Androgen Flare before ADT Is Achieved

**Figure 1:**
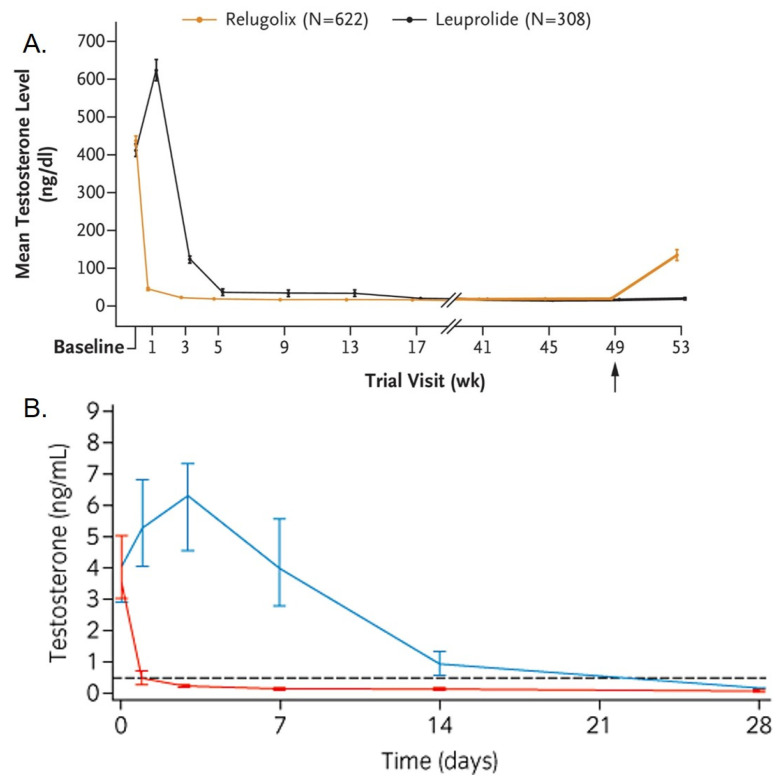
(**A**) Testosterone levels over time after initiation of relugolix (orange) or leuprolide (black) from the HERO trial. Reproduced with permission from the New England Journal of Medicine, Shore, N.D.; Saad, F.; Cookson, M.S.; George, D.J.; Saltzstein, D.R.; Tutrone, R.; Akaza, H.; Bossi, A.; van Veen-huyzen, D.F.; Selby, B., et al., Oral Relugolix for Androgen-Deprivation Therapy in Advanced Prostate Cancer, Volume No. 382, Page No. 2187–2196, Copyright © 2022 Massachusetts Medical Society. (**B**) Median serum testosterone levels in the first month of treatment in a randomized, controlled study of degarelix (red) versus leuprolide depot (blue). Dotted line at 0.5 ng/mL; below this threshold are testosterone castrate levels. Reproduced with permission from the International Journal of Urology: official journal of the Japanese Urological Association. Van Poppel, H.; Klotz, L. Gonadotropin-releasing hormone: an update review of the antagonists versus agonists. 2012, 19, 594–601. Permission was obtained as well from the original publication from which the figure was adapted, BJU international. Klotz, L.; Boccon-Gibod, L.; Shore, N.D.; Andreou, C.; Persson, B.-E.; Cantor, P.; Jensen, J.-K.; Olesen, T.K.; Schröder, F.H. The efficacy and safety of degarelix: a 12-month, comparative, randomized, open-label, parallel-group. 2008, 2.

Long-acting LHRH agonists result in a surge in androgens which starts a few hours after administration and lasts for a few days [15,16]. The HERO study by Shore et al. [13] randomly assigned patients with advanced prostate cancer to daily oral relugolix (LHRH antagonist) or long-acting leuprolide (LHRH long-acting agonist) injections once every 3 months. The authors measured testosterone levels as a function of time from the start of LHRH agonist. At week 2 from the initiation of leuprolide, testosterone levels were approximately 50% higher compared to baseline (Figure 1A). Klotz et al. published a study that evaluated the efficacy and safety of degarelix (LHRH antagonist) versus monthly leuprolide [15,17]. After the initiation of leuprolide, median testosterone levels increased by 65% from baseline by day 3 (median testosterone level 6.30 ng/mL; *p* < 0.001) [17]. Median testosterone levels remained above castrate levels (0.5 ng/mL) until day 21 [17] (Figure 1B). Sasagawa et al. [18] measured serum concentrations of LH and testosterone in 16 patients with advanced prostatic cancer before and after treatment with leuprolide. The increase in relative LH values was noted for 7 days, with a maximum of 345 ± 108% (means± standard error) on day 2 after LHRH analogue injection. For testosterone, elevation of its levels after LHRH analogue application was noted for 7 days, with a maximum of 145 ± 13% on day 2 [18]. Thus, there is a period of approximately 7–15 days of testosterone flare after the initiation of LHRH agonist, which is much more extended in some patients [13], and likely depends on the LHRH type and its dose.

## 3. Antiandrogens Do Not Result in Castration, and There Is No High Level of Evidence to Show That They Protect from Testosterone Flare

Bicalutamide, an antiandrogen, when used as monotherapy for patients with prostate cancer, results in a rise in LH, estradiol, and testosterone levels [19]. Non-steroidal antiandrogens are regarded as a therapeutic option for patients with advanced prostate cancer who wish to retain sexual interest and function [20]. Early Prostate Cancer (EPC) trial randomized patients with localized or locally advanced, nonmetastatic prostate cancer, to bicalutamide 150 mg once daily or to placebo, in addition to standard care, and found a non-statistically significant difference in erectile dysfunction between the two groups [21]. Prostate-specific antigen decreases under treatment with bicalutamide, and that is why it is erroneously called a “castrating” medication, despite that most potent patients continue to maintain potency after the initiation of antiandrogen monotherapy [21].

In patients with a surge in testosterone, as happens during the first 1–2 weeks of LHRH therapy, the benefit of antiandrogens is much more obscure. The idea that antiandrogens can “protect” from testosterone flare was investigated in multiple studies. Oh et al. [22] identified newly diagnosed metastatic prostate cancer patients treated at the Veterans Affairs Hospitals from 2001 to 2004 with LHRH agonists with or without prior antiandrogen therapy. Antiandrogen therapy before LHRH agonist initiation in patients with metastatic prostate cancer was not associated with differences in fractures, spinal cord compression, bladder outlet obstruction, or change in narcotic prescription [22]. Vis et al. [23] reviewed the literature regarding testosterone flare, and found that there is a lack of compelling data showing definite disease progression during the short period of testosterone flare after initiation of LHRH agonist therapy. A more recent review of the literature by Krakowsky et al. found that testosterone flare does not appear to be associated with significantly increased PSA, disease progression, or adverse events, even in men with widely metastatic disease [24]. Testosterone flare after initiation of LHRH could theoretically result in symptomatic progression of prostate cancer, though we do not have a high level of evidence to show that this happens, and thus the role of antiandrogens in preventing these side effects is controversial.

Radiation with bicalutamide therapy in patients with recurrent prostate cancer after radical prostatectomy, showed significantly higher rates of long-term overall survival compared to radiation alone [25]. ADT in the salvage setting showed survival benefit only when goserelin was provided on the first day of radiation, as in GETUG-AFU 16 [26]. Thus, starting LHRH therapy before radiation is not supported by any randomized trial in the salvage setting. Neoadjuvant ADT should not be a substitute for bicalutamide, or provided instead of starting LHRH on day 1 of radiation.

## 4. Androgens Drive Prostate Cancer Cells into Mitosis

**Figure 2:**
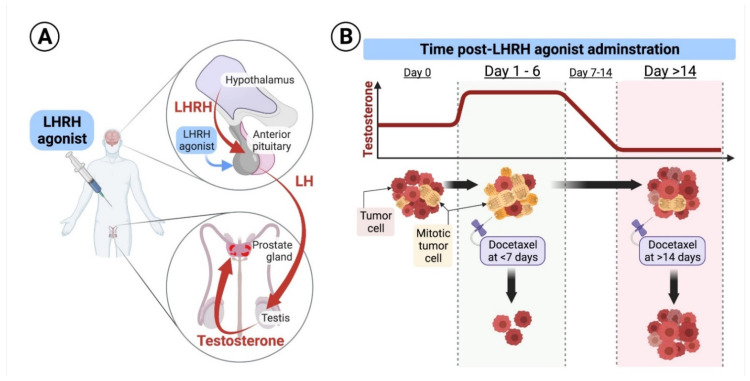
(**A**) Schematic diagram of the LHRH mode of action: Under physiologic conditions, the hypothalamus secretes luteinizing hormone-releasing hormone (LHRH) in a cyclic manner to induce secretion of luteinizing hormone (LH) from the pituitary, which results in secretion of testosterone from the testicles. LHRH agonists used for treatment of prostate cancer are formulated in long-acting and continuously released forms, that are injected subcutaneously and released to the systemic circulation at high levels for protracted periods of times, ranging from 1 to 6 months. Continuously released LHRH agonists mask the cyclic secretion of LHRH from the hypothalamus, leading to a drop in LH secretion from the pituitary and suppression of androgen secretion from the testicles. (**B**) Effect of docetaxel at different times of application: Upon initiation of long-acting formulations of LHRH agonists, testosterone levels surges for few days, and then start declining to castrate levels. Testosterone flare results in increased mitosis of hormone-sensitive prostate cancer cells. Secondary analysis of the CHAARTED trial shows that delivery of the first dose of docetaxel during testosterone flare, at days 1–6 after LHRH initiation, results in better clinical outcomes, compared to more than 14 days when testosterone is at sub-physiologic or castrate levels [33]. Reproduced from Nasser, N.J.; Sun, k.; Scanlon, K.M.; Mishra, M.V.; Molitoris, J.K. Administering Docetaxel for Metastatic Hormone Sensitive Prostate Cancer 1–6 Days Compared to More than 14 Days After the Start of LHRH Agonist is Associated with Better Clinical Outcomes Due to Androgen Flare. Published in Cancers 2022, 14(4), 864; CC BY 4.0.

The androgen receptor pathway is a key driver of prostate cancer progression [27]. Androgen activates the androgen receptor which is critical for survival and proliferation of androgen-sensitive prostate cancer cells [28]. The seminal studies by Huggins et al. [1], and multiple studies published after that, highlighted androgen deprivation as pivotal in the management of advanced prostate cancer and high-risk localized disease [29,30,31]. Androgen deprivation has significant side effects: impotence, hypertension, obesity, and diabetes [32]. Thus, there is a need for medications capable of curing prostate cancer without androgen deprivation. Docetaxel is a chemotherapy that is effective against prostate cancer, and specifically targets cells during cell division. By stabilizing the mitotic spindle, docetaxel induces “mitotic catastrophe” and death of the dividing cancer cells [33,34,35,36,37,38,39,40]. Docetaxel, when given at the beginning of LHRH therapy for patients with metastatic hormone-sensitive prostate cancer (MHSPC), results in significantly longer overall survival than LHRH therapy alone [41,42,43,44]. We recently published [33] a secondary analysis of the CHAARTED trial, which randomized MHSPC patients to ADT alone or ADT plus docetaxel [41]. We showed that by providing the first dose of docetaxel during testosterone flare, at 1–6 days from LHRH initiation, patients could have better clinical outcomes, compared to patients who started docetaxel more than 14 days from LHRH initiation, as testosterone specifically drives prostate cells into mitosis, priming it to cell kill by docetaxel [33] (Figure 2).

## 5. Mitotic Death Is the Dominant Mechanism of Cancer Cell Kill following Radiation

For most cells, death while attempting to divide, that is, mitotic death, is the dominant mechanism of cell kill following radiation [45]. Radiation therapy, similar to treatment with docetaxel, mainly targets dividing cancer cells in mitosis. Combining LHRH and radiation therapy was shown to increase survival of prostate cancer patients with locally advanced disease [46,47], and of patients with a low metastatic burden [48]. The mechanism of synergism between LHRH and radiation is not clear. Here, we present preliminary evidence, based on previous randomized controlled trials, that androgen surge during radiation, rather than its suppression, could be responsible for the enhanced prostate cancer cell kill.

**Figure 3:**
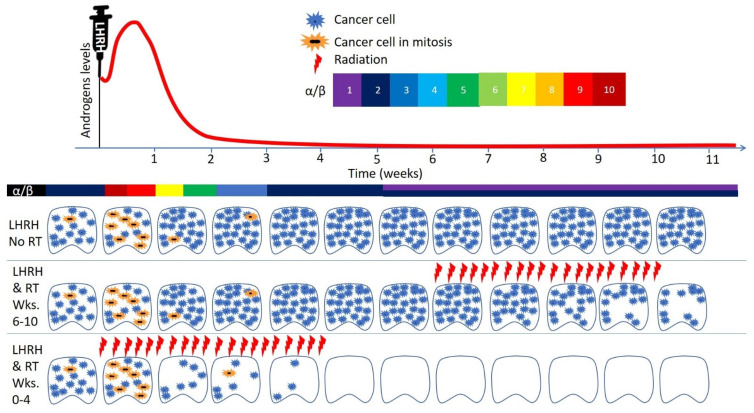
Prostate cancer treatment with luteinizing hormone-releasing hormone (LHRH), without or with radiation. Long-acting, continuously released LHRH initially results in increased production of androgens in the testicles, and secretion of testosterone to the systemic circulation for few days. During the first 7–10 days after LHRH initiation, blood androgen levels increase in what is known as androgen flare. Androgen flare most likely results in a change in the alpha/beta ratio of the prostate, and drives it to high values, most likely of 10 Gy or more. After androgen flare, the alpha/beta ratio drops until a level of approximately 1.5 Gy is reached. During androgen flare, prostate tumor cell division increases. With maintenance of continuous LHRH therapy, androgen deprivation ensues and results in prostate cancer cells becoming less metabolically active, and tumor cell division decreases significantly (top panel, prostate cancer treated with LHRH no RT). When neoadjuvant LHRH is provided, and radiation therapy is delivered 6–10 weeks after LHRH initiation, the number of dividing prostate cancer cells at time of radiation is low, necessitating radiation dose escalation to achieve tumor control (LHRH and RT weeks 6–10), as in RTOG 9413. This is in contrast to providing radiation therapy during androgen flare, when more prostate cancer cells progress to mitosis, priming them to cell kill by radiation, as in EORTC 22863 and RTOG 85–31 (LHRH and RT weeks 0–4).

The linear-quadratic model is used in radiation oncology to estimate tumor control probability and normal tissue complication probability using logistic models [49]. The alpha/beta ratio is the dose where the linear and the quadratic component causes the same amount of cell kill [50]. Rapidly proliferating tumors, such as lymphoma [51] and non-small-cell lung cancer [52], have a high alpha/beta ratios of 10 Gy or more [50]. Prostate cancer has a lower alpha/beta ratio of approximately 1.5–3 Gy [53,54,55,56]. These alpha/beta ratios of the prostate were measured under radiation therapy alone, or in combination with ADT. During androgen flare, the number of mitotic cancer cells increases, and thus the alpha/beta ratio at that time will necessarily be higher than that without androgen stimulation or during androgen deprivation (Figure 3). How high the alpha/beta ratios of prostate during androgen flare is a matter that will need to be investigated radiobiologically, but it probably could reach 10 Gy or even more, as radiation therapy spanning a short period of testosterone flare, as in the Radiation Therapy Oncology Group (RTOG) 8531 trial [47,57] and in the European Organization for Research and Treatment of Cancer (EORTC) 22863 study [46] resulted in significant absolute survival benefits, compared to radiation only, of approximately 10–20% at 10 years of follow-up. This is in contrast to the RTOG 9413 trial in which radiation during testosterone flare was avoided and providing ADT before or after radiation did not result in any significant difference in survival [58].

## 6. Studies Testing the Combination of LHRH Agonist and Radiation

### 6.1. The Radiation Therapy Oncology Group 85–31 Trial

The RTOG 8531 trial [47] was a national prospective randomized trial of standard external-beam irradiation, plus the LHRH agonist, goserelin, which was started in the last week of radiation and delivered indefinitely or until sign of disease progression (arm I), versus radiation alone with hormone manipulation at the time of relapse (arm II). The initial target volume was the whole pelvis, and was treated with 45 Gy. The prostatic boost volume received 20 to 25 Gy, bringing the total prescribed dose to that volume to 65–70 Gy [47]. The 5 and 9 year absolute survival rates were 72% and 62%, respectively, for all patients in arm I; and 50% and 38%, respectively, for all patients in arm II. P value was 0.23 on univariate analysis; but on multivariate analysis, results were statistically significant (*p* = 0.030) [47]. Thus, at 5 and 9 years, there was a, respectively, 22% and 24% absolute difference in overall survival between the two arms. At 10 years, the absolute survival rate was significantly greater for arm I than for the control arm: 49% vs. 39%, respectively (*p* = 0.002) [57].

### 6.2. The European Organization for Research and Treatment of Cancer (EORTC) 22863 Study

**Figure 4:**
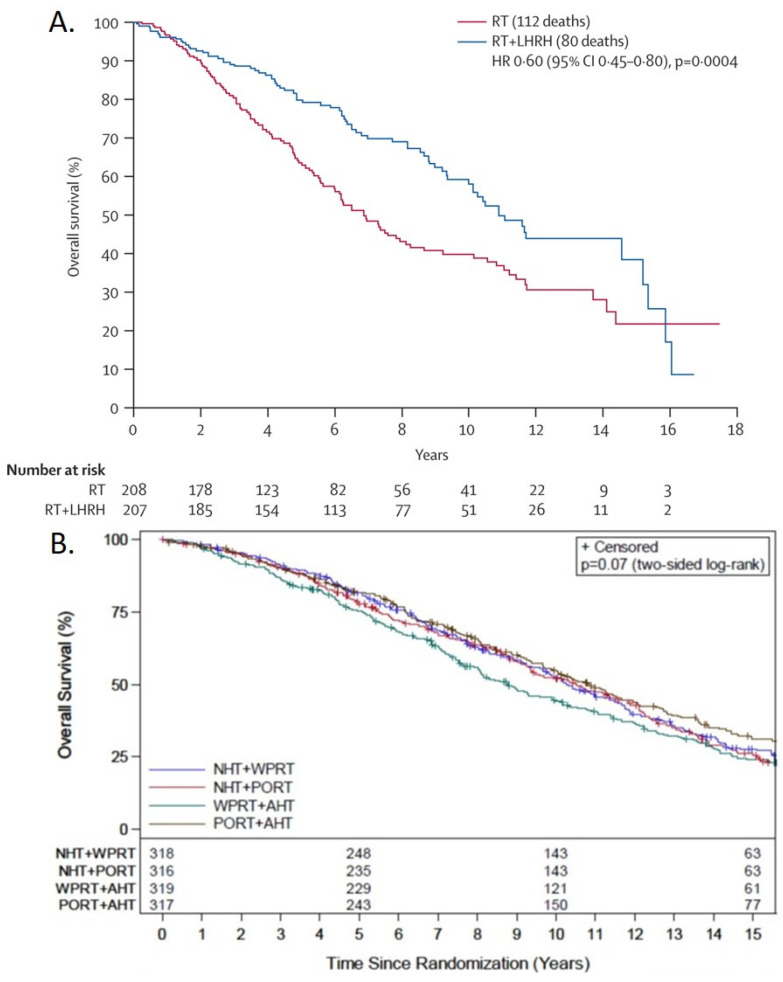
Overall survival in prostate cancer patients with locally advanced disease treated within the (**A**) EORTC trial 22863, which randomized patients to radiotherapy (RT) alone or to RT + luteinizing hormone-releasing hormone (LHRH) starting at day 1 of RT. Reprinted from The Lancet Oncology, Vol. number 11, Bolla, M.; Van Tienhoven, G.; Warde, P.; Dubois, J.B.; Mirimanoff, R.-O.; Storme, G.; Bernier, J.; Kuten, A.; Sternberg, C.; Billiet, I. External irradiation with or without long-term androgen suppression for prostate cancer with high metastatic risk: 10-year results of an EORTC randomised study. 1066–1073, 2010, with permission from Els1evier. (**B**) RTOG 9413 trial randomized patients to neoadjuvant hormonal therapy (NHT) or adjuvant hormonal therapy (AHT) with radiation to prostate only (PORT) or to the whole pelvis (WPRT). Reprinted from The Lancet Oncology, Vol. number 19, Roach, M.; Moughan, J.; Lawton, C.A.F.; Dicker, A.P.; Zeitzer, K.L.; Gore, E.M.; Kwok, Y.; Seider, M.J.; Hsu, I.C.; Hartford, A.C., et al. Sequence of hormonal therapy and radiotherapy field size in unfavourable, localised prostate cancer (NRG/RTOG 9413): long-term results of a randomised, phase 3 trial. 1504–1515, 2018, with permission from Elsevier.

The EORTC 22863 study by Bolla et al. was a randomized, prospective trial comparing external irradiation with external irradiation plus goserelin, which was started on the first day of irradiation and continued for 3 years, and an antiandrogen that was given for 1 month starting a week before the first goserelin injection. The 5-year overall survival was 62% and 78%, respectively (*p* = 0.0002), and the 10-year overall survival was 39.8% and 58.1% (*p* = 0.0004), respectively, in patients receiving radiotherapy alone compared to those allocated to combined treatment—an absolute overall survival difference between the two arms of 16% and 18.3%, at 5 and 10 years, respectively (Figure 4A) [46]. A subsequent study from the EORTC investigated the benefit of prolonged hormonal therapy after radiation and hormonal therapy in patients with locally advanced prostate cancer. All patients received external-beam radiotherapy plus 6 months of LHRH analogue initiated on the first day of radiation, and an antiandrogen agent (flutamide or bicalutamide), initiated 1 week before the start of treatment with the LHRH analogue. After completing 6 months of hormonal therapy, patients were randomized to observation or treatment with the same LHRH analogue but without the antiandrogen for another 2.5 years [59]. The 5-year overall survival was 81% and 84.8% for the short- and long-term hormonal therapy, respectively, a 3.8% survival benefit for the additional 2.5 years of hormonal therapy; an interim analysis showed futility, and the results reached significance only with a post hoc adjusted one-sided alpha level [59]. No long-term results published since the initial report in 2009, and despite that the accrual to the study was completed in 2002 [59]. So, this study, together with EORTC 22863, shows that the main survival benefit for combined therapy is the radiation and the first 6 months of hormonal therapy in which LHRH was provided starting on the first day of radiation.

### 6.3. The Radiation Therapy Oncology Group 8610 Trial

RTOG 8610 was the first phase III randomized trial to evaluate neoadjuvant ADT in combination with external-beam radiotherapy in men with locally advanced prostate cancer [60]. Patients received combined ADT that consisted of goserelin 3.6 mg every 4 weeks and flutamide 250 mg tid for 2 months before and concurrent with RT, or they received RT alone. There was no significant difference in survival between the two groups [60] (Table 1).

### 6.4. The Radiation Therapy Oncology Group 9202 Trial

RTOG 9202 was a phase 3 trial that randomized 1554 patients with locally advanced prostate cancer with PSA < 150 ng/mL, who completed 4 months of goserelin and flutamide, 2 months before and 2 months during RT to a dose of 65 to 70 Gy to the prostate and 44 to 50 Gy to the pelvic lymph nodes, to 24 months of goserelin or no further treatment. Overall survival was not significantly different between the two treatment arms—80.0% vs. 78.5% at 5 years, *p* = 0.73 [61].

### 6.5. The Radiation Therapy Oncology Group 9413 Trial

**Figure 5:**
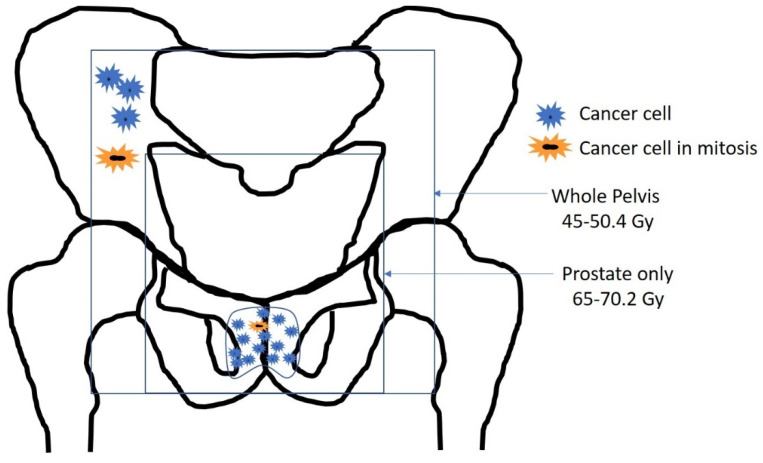
RTOG 9413 trial. Radiation therapy to prostate only (PO) was limited to the prostate and seminal vesicles, with a maximum unblocked field size of 11 × 11 cm to a total dose of 70.2 Gy, 1.8 Gy per fraction per day, 5 days a week. Radiation therapy to the whole pelvis was performed using a minimum unblocked field size of 16 × 16 cm to a maximum central axis dose of 50.4 Gy, followed with an additional 19.8 Gy to the prostate only. Suppression of prostate cancer cell mitosis by neoadjuvant ADT could result in resistance to radiation doses in the range of 45–50 Gy. Delivery of LHRH during radiation, as in RTOG 8531 and EORTC 22863, results in testosterone flare, driving tumor cells into mitosis during the radiation therapy course, rendering them sensitive to a dose per fraction of 1.8–2 Gy, and to cumulative doses in the range of 45 Gy, and leading to higher cancer cell death, and thus increased overall survival.

RTOG 9413 was a 2 × 2 factorial study that tried to prove that better castration at the start of radiation therapy could result in survival benefit compared to providing hormonal therapy after radiation, and that radiation to the whole pelvis is superior to prostate only in patients with locally advanced prostate cancer. All patients received LHRH agonist, goserelin or leuprolide, and an antiandrogen, flutamide, for 4 months. The first group began hormonal therapy 2 months before radiation and continued to receive it during radiation, whereas the other group began hormonal therapy immediately following the completion of radiation [58]. Radiation therapy (RT) was given at 1.8 Gy/fraction to a total dose of 70.2 Gy. Whole-pelvis (WP) RT consisted of a conventional four-field “box” technique with a minimum unblocked field size of 16 × 16 cm to a dose of 50.4 Gy, followed by an additional 19.8 Gy to the prostate. Prostate-only (PO) RT was limited to the prostate and seminal vesicles, with a maximum unblocked field size of 11 × 11 cm to a total dose of 70.2 Gy [62]. The 10 year estimates of overall survival did not differ significantly between the groups (Figure 4B) [58]. This led the authors to conclude that there are sequence-dependent and volume-dependent interactions between hormonal therapy and radiotherapy [58]. Here, we provide an explanation of the potential interaction between the sequence of hormonal therapy and the volume of the radiation fields in the treatment of prostate cancer (Figure 5).

## 7. Testosterone Flare after Luteinizing Hormone-Releasing Hormone Injection Is the Side Effect That Likely Makes Most of the Beneficial Effect When It Coincides with Radiation Therapy for Prostate Cancer

While radiation therapy to a dose of 70 Gy could be sufficient to kill non-mitotic prostate cancer cells, 45–50 Gy could be sufficient to kill mitotic prostate cancer cells. Providing neoadjuvant hormonal therapy, as in RTOG 9413, results in driving tumor cells into mitosis during androgen flare, followed by suppression of tumor cell mitosis when androgen deprivation is achieved, and when radiation is delivered (Figure 3). Thus, the radiation in this arm was delivered while the cells are resistant to radiation. This is in contrast to RTOG 8531 and EORTC 22863, when part of the radiation was delivered during androgen flare, driving prostate cancer cells into mitosis, priming them to cell kill by radiation. Neoadjuvant hormonal therapy failed to provide survival benefit. Recent metanalysis showed that longer extension of total ADT duration in the neoadjuvant setting from 3–4 months to 6–9 months did not result in survival benefit [63], and there was no survival benefit for adjuvant short-term ADT versus long-term ADT at 10 years (66% versus 67%) [63]. This in contrast to the EORTC study that showed modest survival benefit for extending adjuvant ADT from 6 months to 3 years [59]. Thus, the main survival benefit in combining hormonal therapy and radiation is achieved from starting LHRH during radiation, not before or after. Testosterone flare is the most reasonable explanation for the enhanced sensitivity of prostate cancer cells when radiation therapy is combined with LHRH.

## 8. Clinical and Preclinical Data Correlating between Androgen Levels and Prostate Cancer Response

### 8.1. Testosterone and Its Effect on Prostate Cancer

Because of the assumption that ADT is the beneficial part of LHRH agonist therapies, most basic laboratory studies tried to find correlation between ADT and radiation in treating prostate cancer. The seminal study by Polkinghorn et al. [64] showed that treating the castration-resistant prostate cancer cell line LNCaP with increasing concentrations of androgen resulted in increased cell growth except for the highest concentration (10 nmol/L) [64]. The authors found that prostate cancer cells treated with androgen followed by ionizing radiation demonstrated enhanced DNA repair [64]. The authors then used ARN-509, a second-generation antiandrogen, and showed that blocking of the androgen receptor resulted in decreased classical non-homologous end-joining [64]. One explanation for the higher repair after treating cells with androgen is that DNA repair is most active during the S phase of the cell cycle [45].

Morgentaler et al. [65] showed that prostate regrowth following castration as a function of serum testosterone in the rat has a steep initial rise at very low testosterone concentrations, followed by a lower slope of continued rise over an increasing testosterone concentration [65]. Testosterone levels decrease in aging men [66,67], and prostate cancer prevalence increases with age. The relationship between total testosterone levels and prostate cancer has been an area of interest among physicians for decades and conflicting results have been reported [68]. If the model suggested by Morgentaler et al. [65] works in humans, then testosterone surge could have a higher impact in patients with lower baseline testosterone levels when it coincides with radiation.

### 8.2. Supraphysiologic Testosterone Therapy in the Treatment of Prostate Cancer

Bipolar androgen therapy (BAT) was reported in the treatment of castrate-resistant prostate cancer (CRPC) [69,70,71,72,73,74,75,76]. Treating CRPC patients with testosterone cypionate and etoposide on top of androgen deprivation therapy resulted in high rates of PSA and radiographic responses, although all men showed eventual PSA progression [69]. A recent study from the Sidney Kimmel Comprehensive Cancer Center treated 29 patients with CRPC with 400 mg of testosterone cypionate intramuscularly every 28 days concurrent with LHRH agonist or antagonist. Only 14% of patients had PSA response to treatment [77]. Luckily, most patients who progressed on BAT responded to abiraterone or enzalutamide [77]. Supraphysiological androgen levels induce cellular senescence in androgen-sensitive prostate cancer cells and in ex vivo-treated tumor samples [78].

### 8.3. Effect of ADT on Tumor Reoxygenation Potentially Potentiates Response to Radiation

Milosevic et al. showed that androgen deprivation increases prostate cancer oxygen levels, and this might explain the improved patient outcome that has been observed in many clinical trials using LHRH agonist in combination with RT [79].

## 9. Developing LHRH Agonist That Its Main Effect Is Androgen Flare

We are developing species of LHRH medications that have androgen flare as the main clinical effect, while trying to avoid androgen deprivation. To that end, we are reversing some of the steps taken to develop long-acting depot medications, using formulations that are similar to the native LHRH agonist, that have a shorter half-life, and exploring doses much lower than currently used in LHRH depot formulations. These new medications will likely open the door for a new era in the treatment of locally advanced and metastatic prostate cancer, in which castration is no longer the backbone of the treatment. Instead of inducing castration to control prostate cancer, these medications will induce androgen flare and couple it with mitosis-targeting chemotherapies or radiation.

### 9.1. A Short-Acting LHRH Pen or Self-Injecting Device

Docetaxel is usually provided as a short infusion once every 21 days. A LHRH pen is a device that we are exploring for self-injection. The model that we are trying to develop is a very short-acting LHRH agonist that can be self-injected 12–36 h before scheduled chemotherapy; so when the infusion of chemotherapy is delivered, the patient will be in androgen flare. The idea is to repeat the whole process once every 3 weeks. Docetaxel half-lives (mean ± SD) are similar, with weekly and 3-weekly schedules (16.5 ± 11.2 versus 17.6 ± 7.4 h) [80]. Androgen flare could result in higher sensitivity to chemotherapy, and thus lower doses of chemotherapy could be needed to induce the same tumor cell kill achieved during ADT. After completion of chemotherapy, patients with residual disease will need treatment with androgen deprivation therapy.

### 9.2. A Method to Induce Cyclic Fluctuations in LHRH during Radiation Therapy

**Figure 6:**
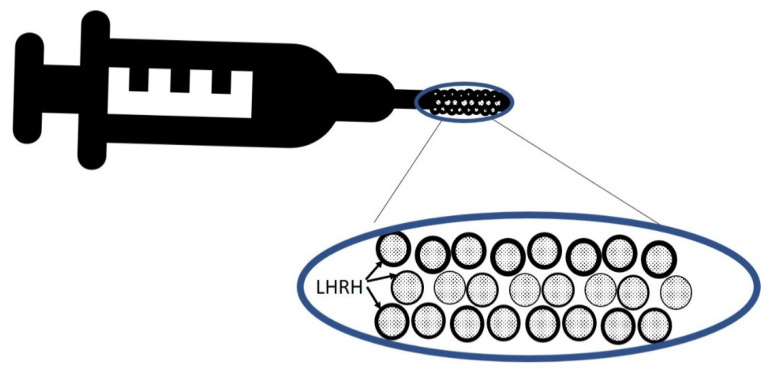
Short-acting LHRH agonists enveloped in dissolvable capsules of varying thickness to allow for cyclic release of LHRH to the circulation.

For locally advanced prostate cancer, we are developing formulations of LHRH agonists with a very short half-life, encapsulated within species of capsules that have different dissolving times, so that LHRH is released in a cyclic manner every several days, so all or most of the radiation course could be delivered during sequential cycles of testosterone flare (Figure 6). At the end of the radiation course, a long-acting LHRH agonist could be delivered to induce ADT. If our hypothesis turns out to be correct, this will result in higher sensitivity to radiation, and will open the door for clinical trials testing of radiation dose de-escalation in the treatment of prostate cancer.

## 10. Potential Use of the Same Concept for Breast Cancer

The main paradigms of treating hormone-sensitive breast cancer are based on molecules that specifically inhibit the estrogen and progesterone receptors, and on chemotherapy. The concept of providing molecules that specifically induce targeted tumor cell division and to couple it with chemotherapy that specifically targets dividing cells was not reported to the best of our knowledge.

### 10.1. Surfing on the Estrogen Wave

The safest way to test this paradigm is within clinical trials that treat premenopausal women with breast cancer. Instead of giving the chemotherapy course on a random day, the timing of chemotherapy courses could be potentially coordinated with the menstrual cycle. The idea is to wait for the estrogen wave, when estradiol approaches peak levels, likely at days 10–14 of the menstrual cycle, and then provide chemotherapy. Similar to surfers who wait for a wave to hang on the surfboard, the timing of chemotherapy, which specifically targets dividing cells, could be tuned to the timepoint at which the body secretes estrogen, that specifically induces cell division of breast cancer cells. This could potentially result in better treatment outcomes. Inducing a larger wave of endogenous estrogen is something that may be considered if simple studies focused on timing of chemotherapies provide promising results. Chemotherapy can induce amenorrhea and thus finding the “estrogen wave” can be challenging after the first courses of chemotherapy are delivered.

### 10.2. Providing Estrogen or Progesterone before Chemotherapy

The other potential way to test this concept in the treatment of breast cancer is through a clinical trial that gives immediate-release estrogen or progesterone before chemotherapy is provided. We are considering the development of an estrogen pen or self-injecting needles that allow delivery of short-acting estrogen and/or progesterone 8–12 h before chemotherapy, to specifically induce breast cancer tumor cell mitosis, and couple it with mitosis-targeting chemotherapies. This combination of hormonal therapy with chemotherapy will most likely need to be coupled with a low-molecular-weight heparin (LMWH), such as enoxaparin, for 2–3 days, to prevent hypercoagulability from the combination of the treatments, and to stop the LMWH before the expected nadir time of thrombocytes, to prevent potential bleeding. If clinical trials show benefit of such combinations, then the next step will be chemotherapy dose de-escalation, to arrive to the minimal effective dose.

## 11. Conclusions

Starting LHRH agonists on the first day (preferable) or last week of radiation therapy for localized prostate cancer, rather than before or after radiation, has the highest level of evidence for survival benefit. Androgen flare could be the “side effect” that provides most of the beneficial effect when it coincides with radiation therapy for prostate cancer. Providing short-acting agonists that specifically induce tumor cell mitosis and coupling it with treatments that target cell mitosis such as taxanes or radiation is a concept that, if proven to be correct, could result in a new era in the treatment of locally advanced and metastatic prostate cancer, in which treatment de-escalation and cure of metastatic disease could become possible.

The current standard of care of hormonal therapy in the United States and Canada resulted erroneously from skipping one clinical trial. Instead of taking the winning arm of the RTOG 8531 trial that showed significant survival benefit when LHRH agonist was given during the last week of radiation, compared to radiation alone [47,57], the researchers chose the winning arm of RTOG 8610, which provided neoadjuvant, concomitant and adjuvant hormonal therapy and radiation, and did not show significant survival benefit compared to radiation alone [60]. RTOG 9413 chose the winning arm of RTOG 8610, which did not show survival benefit, and compared it to fully adjuvant hormonal therapy, rather than providing LHRH during the last week of radiation. No trial compared the winning arms RTOG 8531 and RTOG 8610.

Giving the first dose of LHRH agonist on the first day [46] or last week [57] of radiation showed a clear survival benefit compared to radiation alone. This may be due to testosterone flare or because of other hormones or effects that LHRH agonists induce and we do not know about them yet. The standard of care for prostate cancer patients should be one of the trial arms that showed significant survival benefit when hormonal therapy was combined with radiation as in RTOG 85–31 and EORTC 22863. Because of the similar survival in the control arms of these studies (Table 1), and the higher overall survival in EORTC 22863, starting LHRH agonist on the first day of radiation for patients with high-risk prostate cancer should be the standard of care, until a better standard evolves.

## 12. Patents

The author is an inventor on pending patents filed by the University of Maryland Baltimore on methods to treat cancer by providing medications that induce targeted tumor cell mitosis before providing chemotherapy or radiation, and methods to induce cyclic fluctuations in LHRH and other hypothalamic hormones.

## Figures and Tables

**Table 1:** Clinical trials comparing radiation therapy only to radiation therapy + hormonal therapy.

**RTOG 8531**	**RT + Goserelin Started in the Last Week of Radiation and Delivered Indefinitely**		**RT Only**	
		10 Yrs. survival		
	49%		39%	*p* = 0.002
**EORTC 22863**	**RT + Goserelin Started on the First Day of Radiation and Continued for 3 Yrs.**		**Rt only**	
		10 Yrs. survival		
	58.1%		39.8%	*p* = 0.0004
**RTOG 8610**	**RT + Goserelin 3.6 mg Every 4 Weeks and Flutamide 250 mg Tid for 2 Months before and Concurrent**		**Rt only**	
		10 Yrs. survival		
	43%		34%	*p* = 0.12

## Abbreviations

LH: luteinizing hormone; LHRH: luteinizing hormone-releasing hormone; CRF: corticotropin-releasing factor; MHSPC: metastatic hormone-sensitive prostate cancer; RTOG: Radiation Therapy Oncology Group; EORTC: European Organization for Research and Treatment of Cancer; RT: radiation therapy; WP: whole pelvis; PO: prostate only; NHT: neoadjuvant hormonal therapy; AHT: adjuvant hormonal therapy; PORT: prostate-only radiation therapy; WPRT: whole-pelvis radiation therapy; CRPC: castrate-resistant prostate cancer.
